# Self-Choice Emotion Regulation Enhances Stress Reduction: Neural Basis of Self-Choice Emotion Regulation

**DOI:** 10.3390/brainsci14111077

**Published:** 2024-10-28

**Authors:** Nozomi Imajo, Yutaka Matsuzaki, Akiko Kobayashi, Kohei Sakaki, Rui Nouchi, Ryuta Kawashima

**Affiliations:** 1 Institute of Development, Aging, and Cancer (IDAC), Tohoku University, Sendai 980-8575, Japan; kobayashi.akiko@kochi-tech.ac.jp (A.K.); kohei.sakaki.b4@tohoku.ac.jp (K.S.); ryuta@tohoku.ac.jp (R.K.); 2 Graduate School of Letters, Arts and Sciences, Waseda University, Tokyo 162-8644, Japan; 3 School of Economics & Management, Kochi University of Technology, Kochi 780-8515, Japan; 4 School of Psychological Sciences, University of Human Environments, Matsuyama 790-0825, Japan

**Keywords:** emotion regulation, self-determination, inferior frontal gyrus

## Abstract

Background/Objectives: Opting to perform emotion regulation when facing high-arousal stimuli enhances the reduction in negative emotions. Previous research has indicated that self-choice, that is, personally choosing from multiple alternatives, can improve performance. However, it is unclear whether the emotion regulation strategy chosen among multiple alternatives in daily life enhances stress reduction compared to a forced strategy. This study aimed to reveal the effects of self-choice emotion regulation and its underlying neural basis. Methods: Participants were 40 healthy adults who met the inclusion criteria; they performed self-choice emotion regulation, forced emotion regulation, and no emotion regulation (the control condition) while their brain activity was captured using a functional magnetic resonance imaging scanner. First, the participants were shown a stressful scenario. Secondly, they rated the stress they experienced. Thirdly, they performed self-choice or forced emotion regulation or did nothing. Finally, participants rated their stress level again. Results: Self-choice emotion regulation reduced stress better than forced-choice emotion regulation. The stress reduction was associated with decreases in the activation of the left opercular part of the inferior frontal gyrus. Conclusions: Self-choice can improve emotion regulation, and this effect is likely mediated by the neural efficiency of the left inferior frontal gyrus.

## 1. Introduction

To relieve negative emotions and stress in our daily lives, we employ various strategies. For example, when feeling depressed after making a mistake at work, we might try to reset by thinking that we have learned something from this failure. This thought style is an emotion regulation (ER) strategy called “positive reappraisal”. ER includes all the processes whereby individuals influence their emotions, as well as when and how they experience and express these emotions [1]. ER consists of extrinsic and intrinsic processes responsible for monitoring, evaluating, and modifying emotional reactions, especially their intensive and temporal features, to accomplish goals [2]. It involves changes in the latency, rise time, magnitude, duration, and offset of responses in the behavioral, experiential, and physiological domains [1]. Negative emotions are related to stress [3], so this study focused on stress reduction in ER.

ER can be strongly influenced by motivational factors, as well as other cognitive domains [4]. For example, exercises that participants choose to perform [5], memories that participants choose to memorize [6], and stopwatches that participants choose to use for time measuring [7] produce better results than same strategies when participants are forced to perform, memorize, or use them. This effect of self-choice can be explained in terms of self-determination theory [8], which argues that the need for autonomy is essential for facilitating growth, which is the basis for self-motivation [9]. Therefore, self-choice enhances intrinsic motivation and cognitive performance [8]. One of the factors affecting ER choice, which involves whether ER should be performed and the strategy to adopt, is motivation. A systematic review indicated that the goal of ER (i.e., whether to decrease, increase, or avoid the emotion) motivates ER choice [10].

Recent studies have revealed a positive effect of self-choice on ER. For instance, choosing to inhibit or feel negative emotions can reduce negative emotions better than being forced to inhibit or feel negative emotions after seeing certain images [11]. The authors interpreted this result to mean that the act of voluntarily choosing whether to feel passively or to inhibit facilitated the effect of experienced emotions. Under daily life stress, the effects of the promotion of self-choice ER can be observed. However, previous studies have focused only on the choice of ER performance.

The framework of studies on ER choice involves choosing which strategy to use, namely reappraisal or distraction. Five families of ER strategies were outlined in [12], including cognitive change and attentional deployment. Attentional deployment comprises distraction and rumination, and cognitive change comprises cognitive reappraisal and acceptance [12]. In [13], when the ER goal was “to feel better”, distraction was chosen over reappraisal, unlike when the ER goal was “to perform better” during negative feedback with respect to work situations. Some studies focusing on the intensity of stimuli have shown that people tend to choose reappraisal rather than distraction when seeing low-intensity pictures compared to high-intensity pictures [14,15]. Reappraisal affordance also predicts the choice preference for reappraisal over distraction [16]. Moreover, people who tend to choose reappraisal over distraction exhibit high levels of resilience and well-being [17]. However, if people are in conflict, reappraisal is often not chosen among the various available strategies [18]. From these perspectives, two ER choices, namely reappraisal or distraction, are insufficient. Motivation is enhanced when there are three to five alternatives [8]; therefore, the situation of choosing among five ER strategies depending on the place may maximize the ER effect. This is an important and innovative aspect of this study. We also focused on the cognitive aspect of ER because of its adaptiveness [19].

Numerous neuroimaging studies have been conducted on ER. The inferior frontal gyrus (IFG) is involved in enhancing the effect of ER during ordinary emotion downregulation because its activity reflects the inhibition of motor responses associated with emotional reactivity [20]. The authors of [20] reported that increasing activity in the superior frontal gyrus indirectly leads to decreased activity in the amygdala. In addition, the supplementary motor area (SMA) is involved in the implementation of an internal model to compute the value of emotional regulatory actions and guide behavior [21]. When negative emotions are downregulated by reappraisal, activation of the lateral temporal cortex and modulation of the bilateral amygdala occur [22]. The insula is also involved in the ER network [23].

Adding a self-choice perspective, that is, when participants chose to inhibit their emotions, caused stronger activation in the dorsomedial prefrontal cortex than when they were forced to do so [11]. Refs [24,25] argued that self-choice becomes rewarding, per se, because choosing by oneself can induce one’s confidence. Neuroimaging studies such as that reported in [26] have shown that the caudate, which is involved in reward processing, is related to self-choice. Furthermore, the ventromedial prefrontal cortex (vmPFC) plays an important role in promoting the cognitive performance of self-determined choices [7]. However, no study has compared the brain activation associated with performing a chosen ER strategy among multiple alternatives to that associated with performing a forced strategy. However, since the inhibition of amygdala activation by the vmPFC leads to successful ER [27], self-choice may encourage this effect. If self-choice ER leads to specific brain activation with better stress reduction than forced ER, we suggest that self-choice ER among multiple options is superior to forced ER for mental health. Therefore, we aimed to uncover the neural basis for self-choice ER among multiple ER strategy alternatives.

In this study, we focused on two issues uncovered in self-choice ER. First, we aimed to clarify whether the ER strategy, which was selected by the participants themselves from multiple options, reduced stress levels better than forced strategies. Second, we aimed to elucidate the neural basis of this effect. In this study, we employed cognitive ER strategies [19] for self-choice and forced ER. We expected that a self-choice ER strategy would reduce stress, which is evoked by daily stressful situations, better than forced ER because of the self-choice effect [8]. In terms of brain activation, we expected that the left caudate would be activated when people performed self-choice ER because self-choice contains inherent rewards [28], as well as the activation of brain areas related to ER, including the IFG, vmPFC, and SMA.

## 2. Materials and Methods

### 2.1. Pilot Study

#### 2.1.1. Purpose-Pilot Study

To select which picture and text sets were used and which ER strategy was appropriate for each situation, we conducted a pilot study. This pilot study was designed to identify which strategies were frequently used; therefore, the same procedure as the functional magnetic resonance imaging (fMRI) experiment was adopted.

#### 2.1.2. Participants-Pilot Study

A total of 12 healthy volunteers (8 females; age range, 20 y–50 y) participated in this pilot study. They were not experts in ER and had not been trained in ER at the time.

#### 2.1.3. Stimuli-Pilot Study

We used picture and text sets from our previous study [29] to induce negative emotions in daily interpersonal situations. The study [29] was conducted to clarify the neural correlation of adaptive social behaviors in response to frustrating situations with assessments of causal attribution. The study [29] suggested that a neural response in the right anterior temporal lobe, which is the part that integrates emotional and social information, might integrate the social demands of frustrating situations involving external causality, particularly in adaptive social behavior. We used these frustrating situations as stressful stimuli.

We assessed ER strategies using the Cognitive Emotion Regulation Questionnaire [19,30], which measures the tendency to use ER strategies. Positive cognitive ER includes the following five strategies: (i) positive reappraisal, (ii) putting into perspective, (iii) acceptance, (iv) positive refocusing, and (v) refocus on planning. (i) Positive reappraisal is defined as attaching a positive meaning (e.g., I think I can learn something from the situation). (ii) Putting into perspective is defined as playing down the seriousness (e.g., I think that other people go through much worse experiences). (iii) Acceptance is defined as accepting what you have experienced (e.g., I think that I have to accept the situation). (iv) Positive refocusing is defined as thinking about joyful and pleasant things (e.g., I think of nicer things than what I have experienced). (v) Refocus on planning is defined as thinking about how to handle negative events (e.g., I think of what I can do best).

#### 2.1.4. Task-Pilot Study

The appropriateness of each of the 19 positive ER strategies and their corresponding stimuli were evaluated by a rating on a seven-point Likert scale for use in the choice and forced conditions of the fMRI experiment. There was no “winning” strategy in each scenario. The stress level in each situation was rated to ensure that the stress levels were the same. Five sentences were created for each scenario. Volunteers also rated how appropriately each sentence expressed the scene for use in the control condition of the fMRI experiment.

### 2.2. fMRI Experiment

#### 2.2.1. Purpose-fMRI Experiment

An fMRI experiment was conducted to clarify the effects of self-choice ER. The study was conducted in accordance with the Declaration of Helsinki, and the protocol was approved by the Ethics Committee of Tohoku University (2018-1-272) on 23 July 2018. Informed consent was obtained from all subjects involved in the study.

#### 2.2.2. Participants-fMRI Experiment

We recruited 52 healthy students from Tohoku University, Japan. All the participants were right-handed native Japanese speakers. We confirmed that they had no history of neurological or psychiatric disease and had normal or corrected-to-normal vision. Twelve participants who showed a higher NIMH Center for Epidemiologic Studies—Depression Scale (CES-D) score [31] than the cutoff (16 points) did not participate in our main fMRI experiment. The reason for using the CES-D score is that it indicates the extent to which the participants felt depressed. This study included the presentation of stressful stimuli; therefore, it was difficult for people with depression to participate. This exclusion criterion was also based on previous studies (e.g., [32]). Finally, we analyzed data from 40 healthy university students (22 females; mean age, 22.03 ± 1.37 years old; age range, 20–27).

#### 2.2.3. Stimuli-fMRI Experiment

Based on the results of the pilot study, we used 36 picture and text sets from [29], which resulted in the same arousal levels and cognitive ER strategies [19,30].

#### 2.2.4. Measures-fMRI Experiment

For the behavioral data, we used the stress level, rated on a seven-point Likert scale, and the subjective effectiveness of the strategy, rated on a seven-point Likert scale. High rating scores indicate strong feelings. This single-item measure is the standard in ER choice research, so it has been used in previous studies of self-choice emotion regulation strategies (e.g., [11,33]) to measure participants’ intensity of emotion. Therefore, we followed this method. Using brain imaging, we recorded fMRI data during the task.

#### 2.2.5. Task-fMRI Experiment

**Figure 1:**
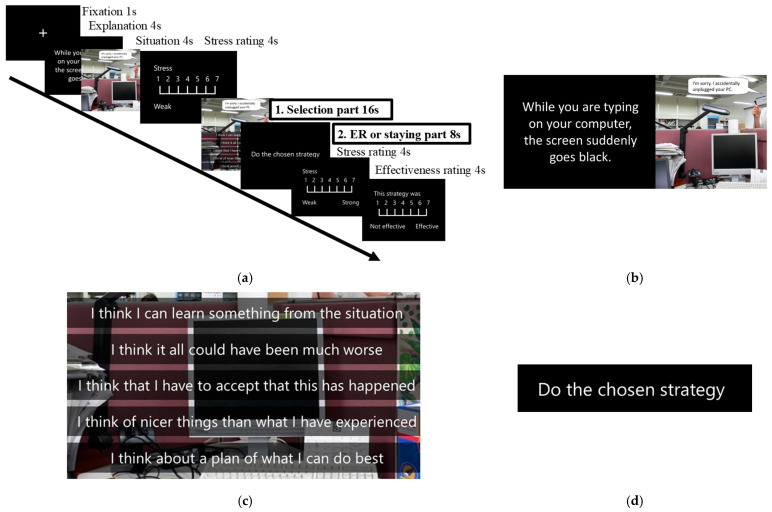
fMRI task overview. (**a**) During the fMRI task, after presenting a fixation cross for 1 s and explaining the process, pictures of a stressful situation depicting a person saying something stressful were presented for 4 s each. After rating their stress using a seven-point Likert scale, participants were instructed to choose one text among the alternatives corresponding to the conditions for 16 s (selection part). Then, participants performed the ER or staying part for 8 s. Next, they were asked to rate their stress level again, as well as the effectiveness of the strategy under the choice and forced conditions, using a seven-point Likert scale for each 4 s. (**b**) A sample of explanations and pictures of a stressful situation [29]. (**c**) Text samples in each part. In the selection part, participants had to choose one of five kinds of emotion regulation strategy [19,30] to use in the situation shown in the choice condition. Participants had to select one predominant (star-marked) strategy in the forced condition. Participants had to choose one sentence among five situations explaining the alternatives in the control condition. (**d**) In the ER or staying part, participants performed the emotion regulation strategy they chose in the selection part of the choice condition or the forced strategy in the selection part of the forced condition. Participants only remained silent, doing nothing, in the MRI scanner during the ER or staying part in the control condition.

Each trial consisted of two parts (Figure 1). In each trial, we presented a scenario describing daily stressful interpersonal situations using pictures and text. After reading the text explaining the situation for 4 s, participants were presented with a picture of the scene for 4 s. Next, they were asked to rate how strongly they felt stressed within 4 s using a seven-point Likert scale.

1. Selection part: After the stress-level rating, participants were presented with five text alternatives and were asked to choose one for 16 s (selection part). We provided five options because a meta-analysis reported that self-choice had the largest effect when participants were provided with three to five options [8]. We set three conditions–choice, forced, and control–each comprising 12 stimuli. The order of the conditions (three conditions) and stimuli (three sets, each containing 12 stimuli) was counterbalanced using a three-Latin-square design. In the choice condition, participants chose one ER strategy from the five strategies mentioned above, i.e., positive reappraisal, putting into perspective, acceptance, positive refocusing, and refocus on planning. In the forced condition, the participants were instructed to choose a predetermined (star-marked) ER strategy from five sentences using the same strategy. We selected a strategy for use under the forced condition based on the results of our pilot study. In the pilot study, we confirmed that the strategies used in the forced condition yielded the highest appropriate scores for each situation. Therefore, the types of strategies used differ slightly. The use frequency of each type of strategy was as follows: refocus on planning, 67.48%; acceptance, 29.62%); and positive reappraisal, 2.90%. In the control condition, we asked participants to choose one sentence to explain the situation selected in the pilot study. This decision-making task included the understanding of each circumstance but did not include the emotional process.
2. ER or staying part: After selection, the participants performed the ER strategy or stayed silent for 8 s (ER or staying part). In the choice condition, the participants performed the ER strategy that they chose to use during the selection part. In the forced condition, participants performed the predominant ER strategy. The participants were instructed to perform putting into perspective and acceptance by repeating the strategy sentences in their minds. Participants were asked to perform positive reappraisal, positive refocusing, and refocus on planning by reading strategy sentences once, thinking about how to deal with each situation, then repeating each idea in their mind. In the control condition, participants did nothing silently in the MRI scanner during the ER or staying part. Therefore, in the control condition, participants did not perform ER. After the ER or staying part, the participants were asked to rate their stress level again, as well as the effectiveness of the strategy, in both the choice and forced conditions using a seven-point Likert scale.

#### 2.2.6. General Procedure

Before the day of scanning, first, we explained the experimental procedure in detail for 30 min in order to acquire informed consent, and we checked the severity of depression in all participants by having them answer a 35 min questionnaire. Secondly, we explained how to apply positive ER strategies only to those with a CES-D score lower than the cutoff, and the participants practiced positive ER strategies until they felt sure they could accomplish them for 15 min. Finally, we explained the details of the task, and participants practiced a few task sets and were asked whether they understood the task and how to perform ER strategies. If they answered in the affirmative, we considered that “they could accomplish the task”. Each explanation and practice session lasted approximately 20 min. The interval between the practice day and the day of fMRI scanning was approximately nine days (*SD* = 12.01). On the day of the fMRI scan, we confirmed that the participants still had low levels of depression. Before scanning, the participants practiced the task again for ten minutes.

#### 2.2.7. Brain Imaging Data Acquisition

During the task, T2*-weighted images (repetition time = 2000 ms, number of slices = 32, flip angle = 50°, slice thickness = 3 mm, slice gap = 0.5 mm, FOV = 192 mm, and matrix = 64 × 64) covering the whole cerebrum were acquired using a gradient echo-planar imaging sequence with an Achieva 3.0T Quasar Dual (Philips, Best, Netherlands) MR scanner. The following fMRI preprocessing procedures were performed using Statistical Parametric Mapping (SPM12) software (r7219, Wellcome Department of Imaging Neuroscience, London, UK) and MATLAB R2008a (Mathworks, Natick, MA, USA): realignment of head motion, correction for slice timing, spatial normalization to Montreal Neurological Institute space, and smoothing using a Gaussian kernel of 8 mm full width at half maximum, according to previous research (e.g., [32]).

All task images were rear-projected onto a semi-lucent screen that could be viewed by the participant via a mirror attached to the MRI head coil. The visual angle was <5°. We recorded participants’ responses by having them press a button. We developed and presented these stimuli using PsychoPy 2 (version 1.90.2, Open Science Tools Ltd., Nottingham, UK, 2018-2019; [34]).

#### 2.2.8. Data Analysis

1. Behavioral data analysis: First, we calculated the frequency of use of each strategy in the choice condition. Secondly, we performed a linear mixed-model analysis to examine whether the degree of stress after the ER or staying part differed by condition. The model was a random-intercept model with stress level as the objective variable, condition (choice, forced, or control condition) and time (pre- and post-ER or staying) and their interaction terms as explanatory variables, and the subject as a random effect. To test the hypothesis that the stress level after ER or staying would be the lowest in the choice condition, we compared the choice vs. forced condition and the choice vs. control condition with corrections for multiple comparisons using Holm’s method. Thirdly, we tested whether the subjective effectiveness of ER strategies differed across conditions. We used a linear mixed model of a random intercept in which the objective variable was subjective effectiveness, the explanatory variable was the condition, and the random effect was the subject. Because subjective effectiveness was rated only in the choice and forced conditions, two conditions discounted the control condition. We used the “lmerTest” and “ppcor” R packages (version 3.5.3) for statistical analyses of behavioral data. The significance threshold was set at *p* < 0.05.
2. fMRI data analysis: To distinguish between brain activation related to choosing a strategy and that relates to its execution, we performed two types of first-level analyses. First, to identify the brain regions involved in choosing a strategy, we used a general linear model (GLM) to investigate the blood oxygen level-dependent (BOLD) signal during the target periods (16 s for the selection part) at a first level. The design matrix for the first-level analysis contained three regressors for the conditions (choice, forced, and control) and six motion regressors. Contrasting images were generated for each condition. In the second-level analysis, we examined the brain regions involved in ER, comparing choice and control conditions by performing *t*-tests on beta maps of contrasts. Secondly, to determine the brain regions involved in ER, we applied a GLM to model the BOLD signal during the target periods (8 s for the ER or staying part) in the first-level analysis. The design matrix for the first-level analysis contained three regressors for the conditions (choice, forced, and control) and six motion regressors. Contrasting images were generated for each condition. For the second-level analysis, a flexible factorial design was applied to compare the brain activation of regions involved in ER relative to the control condition. We examined brain regions that showed greater activity in the forced and choice conditions than in the control. In addition, to examine the brain regions specifically involved in the self-choice ER strategy, we searched for brain regions that showed greater activity in the choice condition than in the forced condition. To determine the common brain regions between self-choice and forced ER, we conducted a conjunction analysis at the voxel level. The statistical threshold was set at *p* < 0.05, and family-wise error was corrected at the voxel level. Finally, we examined the relationship between brain activity related to self-choice ER strategies and stress reduction. For these analyses, we calculated the beta values of the voxels surviving the subtraction of brain activation in the control condition from that in the choice condition. We evaluated the stress reduction level as the difference between the stress levels recorded pre and post ER in the choice condition. We then performed a partial correlation analysis, in which the above-mentioned beta values and stress reduction levels were analyzed. Similarly, we tested the partial correlation between the beta values of the forced condition and the stress reduction level. We included the pre-ER stress values as covariates of no interest. We used the SPM Neuromorphometrics atlas for all analyses.

## 3. Results

### 3.1. Behavioral Data Results

#### 3.1.1. Frequency of Use of Each ER Strategy in the Choice Condition

We investigated the most frequently used strategy in our sample. The selection rates for the ER strategies in the choice condition were 36.75% for refocus on planning, 28.29% for acceptance, 17.15% for positive reappraisal, 13.36% for putting into perspective, and 4.23% for positive refocusing.

#### 3.1.2. Stress Reduction

**Figure 2:**
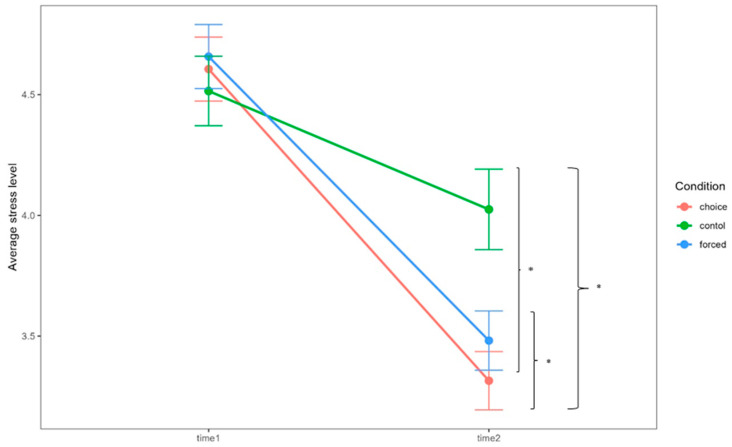
Stress-level ratings in the choice, forced, and control conditions before and after emotion regulation or staying. Time 1 is before emotion regulation (or staying), and time 2 is after emotion regulation (or staying). Error bars indicate standard errors. * indicates significant differences in *p* < 0.05.

To examine the differences in stress reduction levels among the conditions, we performed a linear mixed-model analysis (Figure 2). The results revealed significant main effects of time (*F* (1, 2651.1) = 316.266, *p* < 0.0001), condition (*F* (2, 2651.9) = 11.708, *p* < 0.0001), and the time-by-condition interaction (*F* (2, 2651.1) = 19.970, *p* < 0.0001). Post hoc analyses showed that the stress level before ER did not significantly differ between conditions (choice–forced: *t* (2651.5) = −0.7890, *p* = 0.43, standardized *β* = −0.0451; choice–control: *t* (2651.6) = 0.7265, *p* = 0.47, standardized *β* = 0.0415; control–forced: *t* (2651.4) = −1.5162, *p* = 0.13, standardized *β* = −0.0866). After ER or staying, the stress level in the choice condition was significantly lower than that in the forced (*t* (2651.5) = −1.98, *p* = 0.048, adjusted *p* = 0.048, standardized *β* = −0.113) and control (*t* (2651.6) = −7.53, *p* < 0.0001, adjusted *p* < 0.0001, standardized *β* = −0.4303) conditions. The comparisons of each condition for time 1 and time 2 are shown in Table S1.

#### 3.1.3. Subjective Effectiveness of the Used Strategy

The linear mixed-model analysis showed a significant main effect of condition. The subjective effectiveness in the choice condition was significantly higher than in the forced condition (*t* (1307.5) = 4.075, *p* < 0.0001, *β* = 0.1085).

### 3.2. fMRI Data Results

#### 3.2.1. Brain Activation While Choosing a Strategy in the Choice Condition

First, we examined the brain regions activated during the choice condition in the selection part. The following voxels showed significantly greater activation in the choice condition than in the control condition: the left lingual gyrus, left cuneus, bilateral frontal pole, left caudate, right superior frontal gyrus, and left middle frontal gyrus (Table 1).

#### 3.2.2. Brain Activation During Performance of Forced ER Strategies

**Figure 3:**
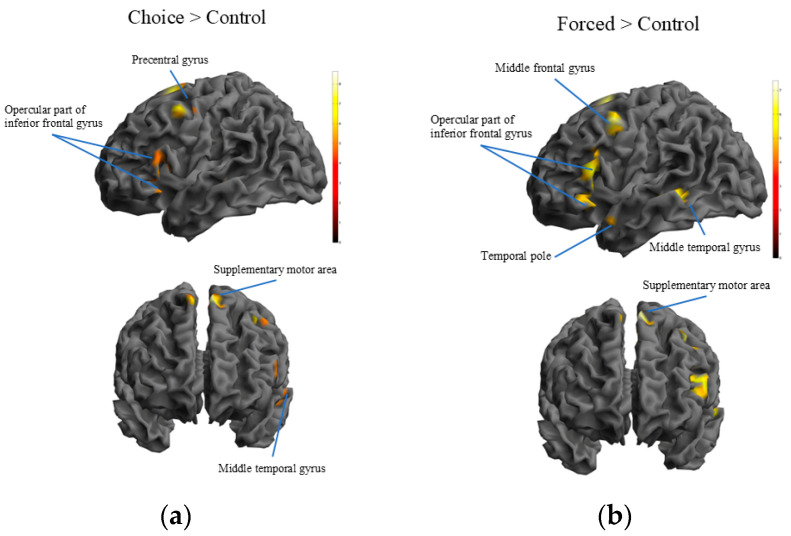
Family-wise error corrected (voxel-level) brain activation during self-choice or forced emotion regulation. The statistical threshold was set at *p* < 0.05. The color bar indicates the *t* value of each area. (**a**) Subtraction of control from choice condition. (**b**) Subtraction of control from forced condition.

Secondly, we examined the brain regions activated under the forced condition during ER. The following voxels showed greater activation in the forced condition than in the control condition: the left SMA, left middle frontal gyrus, left opercular part of the IFG, left frontal operculum, left middle temporal gyrus (MTG), left fusiform gyrus, left temporal pole, and left caudate (Table 2; Figure 3b).

#### 3.2.3. Brain Activation While Performing Self-Choice ER Strategies

The following regions showed significantly greater activation in the choice condition than in the control condition during ER or staying: the left SMA, left precentral gyrus, left frontal operculum, left opercular part of the IFG, left MTG, and left caudate (Figure 3a; Table 3).

Next, we examined the brain regions showing greater activity in the choice condition than in the forced condition. No voxels survived after family-wise error correction at the voxel level. However, activation in the right supramarginal gyrus, right superior parietal lobule, and left postcentral gyrus survived at an uncorrected threshold of *p* < 0.001 at the voxel level (Table 4). We also examined the brain regions showing lower activation in the choice condition than in the forced condition; activation of the bilateral calcarine cortex and bilateral fusiform gyrus survived (Table 5).

#### 3.2.4. Shared Brain Activation Between Self-Choice and Forced ER

To examine the shared activation of brain areas between choice and forced conditions, we performed a conjunction analysis. The following voxels were commonly activated between choice and forced ER during the ER or staying part: the left SMA, left middle frontal gyrus, left opercular part of the IFG, left frontal operculum, left MTG, and left caudate (Table 6). There was a difference mainly in the percentage of refocus on planning used between the forced and choice conditions. Therefore, we also analyzed a model considering the number of selecting the refocus on planning as a covariate. However, the results did not differ much from those obtained when not including this covariate (Tables S2–S5). Therefore, in the following, we describe the results without a covariate.

#### 3.2.5. Correlated Brain Regions Between Self-Choice ER and Stress Reduction

**Figure 4:**
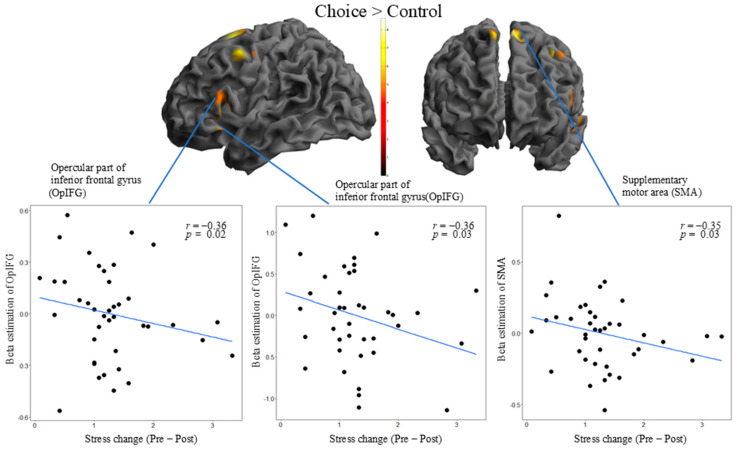
Family-wise error-corrected (voxel-level) correlation of brain activation in self-choice emotion regulation with stress changes. The statistical threshold of each area was set at *p* < 0.05. The color bar indicates the *t* value of each area. The *X* axis indicates the stress-change score. Larger changes indicate stronger stress reduction. The *Y* axis indicates the beta estimation value of each area. We used Pearson’s partial correlation.

We conducted a correlation analysis of the relationship between brain activity during ER depending on self-choice or forced ER strategies and the amount of stress reduction. The relationship between beta values in voxels that were statistically significant in the choice–control and forced–control comparisons and stress values calculated by subtracting post ER from pre ER was tested using partial correlation coefficients, controlling for stress values before ER. In the choice–control comparison, both the left SMA voxel (x = −6, y = 10, z = 64, partial *r* = −0.35, *p* = 0.03) and that of the opercular part of the left IFG (x = −48, y = 14, z = −6, partial *r* = −0.36, *p* = 0.03; x = −50, y = 20, z = 10, partial *r* = −0.36, *p* = 0.02) were significantly negatively correlated with stress values (Figure 4).

## 4. Discussion

This study examined the effect of self-choice ER on stress and its underlying brain mechanisms. This study has four main findings. First, at the behavioral level, self-choice ER reduced stress better than forced ER, supporting our expectation. Secondly, we found shared brain activation between self-choice and forced ER, including in the left opercular part of the IFG. Thirdly, we found that weaker activation of the left opercular part of the IFG and SMA was associated with higher stress reduction only in self-choice ER. Fourthly, we observed significant caudate brain activation when choosing the ER strategy. The results of the brain imaging analysis were partly consistent with our expectations. The reason for this partial consistency might have occurred, to some degree, because of a carry-over of expectation.

Participants who chose ER strategies reduced stress better than those who could not choose strategies in our task. Our findings are consistent with those of previous studies, such as [11]. According to [11], if we choose to inhibit negative emotions and perform inhibition when we can choose either to perform inhibition or do nothing, better regulation occurs than under forced inhibition. Our results correspond to previous work because self-choice ER resulted in a better reduction than forced ER. This result might also support the effect of flexible ER, which is defined as the ability to effectively regulate emotions by applying different ER strategies (chosen from a broad repertoire) in different situations depending on their features, including context-related features—in other words, being adaptive to situation characteristics, as reflected in one’s strategy choice [35]. Because motivation is considered a function of the expectation of success [36], an expectation of the successful effect of ER strategies could reduce stress. Specifically, revealing that self-choice ER, chosen among multiple alternatives, has a larger effect than forced ER is a novel finding of this study. We used a single-item measure for stress in this study. The single-item measure is well-suited for fMRI tasks because fMRI studies need a regularity of time and a short duration for participants to answer questions in experiments [37]. Therefore, several previous fMRI studies of self-choice ER have used a single-item stress measure. Nevertheless, the validity and reliability of this scale requires further consideration.

While performing self-choice ER, we observed significant activation of the SMA, IFG, MTG, and caudate. This result was consistent with that of a previous study (e.g., [23]). During forced ER, we observed significant activation of the SMA, middle frontal gyrus, IFG, MTG, and fusiform gyrus.

Conjunction analysis showed that the left opercular part of the IFG was activated during both self-choice and forced ER, suggesting that the left IFG is important for ER, which is consistent with [38], the authors of which considered that the association of the left IFG with the MTG was due to the habitual use of reappraisal, supporting the goal-driven control of subjective emotion. The authors also reported that the left IFG participates in emotional performance monitoring. During self-choice or forced ER strategies, participants actively regulated the induced negative emotions, including by monitoring their own emotions. Focusing on one’s own emotions might have evoked significant activity in the IFG in both the choice and forced conditions. We also found significant activation of the caudate in both conditions. Our findings are consistent with those of [39], the authors of which reported that the caudate was involved in recovery from discomfort. Our task described daily stressful situations, and we asked participants to regulate the negative emotions evoked by the scenarios. Significant activation of the caudate in the choice and forced conditions may reflect the process of evoking and regulating negative feelings. In addition, we observed significant activation of the SMA. Our results support the findings of [23], which showed SMA activation regardless of the type of regulation strategy used. These common areas of activation are located in the left hemisphere. Supporting the ER brain network in the left part plays the role of reappraising negative emotions and language processing [40]; thus, these results suggest that performing cognitive ER involves significant thinking with language by repeating strategies and reappraising circumstances, which people face positively, regardless of strategy

Our correlation analyses revealed that when stress was substantially reduced, the IFG and SMA were less activated. This result may be explained by neural efficiency, whereby performance improvements are associated with decreased brain activation [41]. The IFG plays an important role in performing ER [38]. Given that the IFG and SMA are involved in ER, those who could self-choose ER and reduce their stress levels required less activation of the regions because of their sophistication in ER.

Nevertheless, our findings do not fully support our prediction. We hypothesized that some specific regions would be involved in self-choice ER. However, we did not find significant differences in brain activation between the choice and forced conditions. Although the results were uncorrected, we found that the supramarginal gyrus and superior parietal lobule showed greater activation in the choice condition than in the forced condition. The supramarginal gyrus and superior parietal lobule are associated with visual and spatial attention [42,43]; thus, this might indicate stronger attention to the task. Activation of the superior parietal lobule when choosing whether to perform ER has also been reported (e.g., [33]). Accordingly, the specific activation of the supramarginal gyrus and inferior parietal lobule during the choice condition indicated that participants might have paid more attention to the stressful situation, driving them to perform self-choice ER strategies rather than forced ER strategies.

In contrast, the fusiform gyrus and calcarine cortex showed greater activation in the forced condition than in the choice condition. This might be due to differences in the strategies used. In the forced condition, participants were often compelled to use the “refocus on planning” strategy. On the other hand, in the choice condition, “refocus on planning” was chosen on 36% of occasions. Thus, the fusiform gyrus, which is related to visual processing of language [44], and the calcarine cortex, which is related to visual images [45], could be related to the strategy of refocus on planning.

In addition, we observed significant brain activation in the caudate, frontal pole, lingual gyrus, right superior frontal gyrus, left middle frontal gyrus, and cuneus during ER choice. The caudate codes for expected reward magnitude [46]. In the context of our study, given the self-determination theory, self-choice ER, itself, seems to include a reward process. In the choice condition, participants could choose one strategy to regulate their negative emotions. The self-determination theory argues that deciding what to do based on one’s own thoughts is rewarding. The activation of the caudate during the choice condition might reflect the expectation of a reward, which includes personally choosing what to do and succeeding in stress reduction. Other activated regions were included in the neural network of cognitive ER [47]. Accordingly, the activation of these regions reflected the consideration of strategies with the expectation of successful stress reduction. This result indicates that when people face situations of interpersonal stress in daily life, they carefully consider which strategy is appropriate, with the expectation of self-choice strategy success.

Our study has eight main limitations. First, we aimed to reveal the effect of self-choice ER on stress reduction in daily stressful situations; therefore, we employed previously developed scenarios [29]. It is possible that we did not evoke stress in the participants’ own experiences. This is still insufficient because these stresses are not realistic enough, owing to the use of imagination in each stressful scenario. However, several reports have indicated that reappraisal after distress and negative emotions is negatively correlated with daily life [48]. Future research should uncover whether the effect of self-choice on stress reduction is enhanced when participants perform self-choice ER strategies in their daily stressful situations. Secondly, we only considered the immediate effect of self-choice ER. However, the regulatory effect has been shown to last for ≥1 week in the ventromedial prefrontal cortex [49]. Therefore, a longitudinal fMRI study is required to elucidate the long-term effects of self-choice ER. Thirdly, we examined the effect of self-choice ER on healthy university students. We excluded university students who had depressive symptoms, so we could only generalize our results to non-depressed undergraduates. Future studies should examine whether self-choice ER strategies reduce stress in both clinical and subclinical populations. For example, people with subclinical paranoia used the cognitive ER strategies of blaming others and catastrophizing habitually, and paranoia scores were predicted by self-blaming [50]. Thus, the enhancing effect of self-choice ER may be different between subclinical and healthy people. Other symptoms such as anxiety are also related to ER according to previous research. For example, anxiety is predicted by less use of positive reappraisal [51]. Therefore, individual differences in anxiety tendency could be considered in future studies. Fourthly, we could not identify the specific activation in the choice and forced conditions because we did not find a significant difference in brain activation between these conditions. Our behavioral results showed significant expectations of the effectiveness of stress reduction only in self-choice ER. Although we used a within-subject experimental design and counterbalanced it, this expectation might be a carryover from the choice to the forced condition. A between-subject design could help detect differences in brain activation between self-choice and forced strategies. Moreover, the proportions of “refocus on planning” strategies used were so discrepant that the difference between self-choice and forced ER might not be displayed in brain imaging results. Reappraisal and distraction are both cognitive ER; however, their neural bases differ. For example, distraction decreases amygdala activation more than reappraisal [52], and the orbitofrontal cortex is especially activated during reappraisal [53]. From these perspectives, in our study, the proportion of strategies used differed significantly between the choice and forced conditions such that the brain imaging contrast of each condition might have been reduced. We only asked about the participants’ subjective sense of accomplishment in performing ER; therefore, the difference in the ability to perform ER between participants might have also influenced the results. The proportion of ER strategies used should be equalized, and an objective ER ability should be obtained in future studies. Fifthly, in this study, we revealed the effect of self-choice ER but could not focus on the choice of the most appropriate strategy for each situation. Future studies are needed to examine differences in effects between good and not-good choices or which individual differences lead to the skills associated with choosing optimal ER strategies depending on circumstances. Sixthly, we did not ask participants in the control condition if they really did nothing. We also did not ask participants in the forced condition if they performed a forced emotion regulation strategy adequately. Such a manipulation check is needed in future studies. Seventhly, we could not consider the probability of consistency in choosing strategies. Some studies have pointed out that emotion regulation is affected by a belief in the malleability of emotions and that this tendency is observed in children (e.g., [54]). Such participant beliefs might influence whether self-choice emotion regulation becomes flexible or rigid; therefore, this belief should be taken into account in future studies. Finally, we only tested stress. In daily life, we have to regulate various negative emotions, such as anger and sadness. Therefore, in future research, whether self-choice strategies are effective for such negative emotions should be examined.

## 5. Conclusions

This study revealed that self-choice ER reduced stress better than forced ER. We found a significant negative correlation between stress reduction and the IFG during self-choice ER, suggesting an important role of the opercular part of the left IFG in self-choice ER.

## Figures and Tables

**Table 1:** Brain areas with significantly greater activation in the choice than control condition during the selection part.

Area	Hemisphere	*t* Value	MNI Peak Coordinates			*p* Value	*k*
			x	y	z		
Lingual gyrus *	L	8.35	−10	−78	0	<0.001	2201
	L	8.21	−10	−70	−6	<0.001	
Cuneus	L	7.61	0	−80	22	<0.001	
Frontal pole	L	5.27	−12	64	2	0.011	12
Frontal pole	R	5.18	10	62	2	0.014	19
Caudate	L	4.90	−10	16	12	0.036	2
Superior frontal gyrus	R	4.85	18	50	8	0.042	1
Middle frontal gyrus	L	4.82	0	50	−8	0.047	1

* Family-wise error-corrected (voxel-level), voxel-by-voxel analysis at *p* < 0.05. Hemispheres are indicated as L = left and R = right. MNI, Montreal Neurological Institute.

**Table 2:** Brain areas with significantly greater activation in the forced than control condition during the ER or staying part.

Area	Hemisphere	*t* Value	MNI Peak Coordinates			*p* Value	*k*
			x	y	z		
Supplementary motor area *	L	7.97	−6	12	62	<0.001	764
		6.45	−6	20	50	<0.001	
		5.70	−4	24	40	0.002	
Middle frontal gyrus	L	7.31	−46	4	52	<0.001	307
Opercular part of the inferior frontal gyrus	L	6.52	−50	20	18	<0.001	570
Frontal operculum	L	5.60	−50	18	−6	0.003	
Opercular part of the inferior frontal gyrus	L	5.45	−50	20	4	0.006	
Middle temporal gyrus	L	5.90	−52	−38	−4	0.001	215
		5.02	−54	−24	−8	0.025	
Fusiform gyrus	L	5.77	−30	−44	−14	0.002	23
Temporal pole	L	5.17	−48	10	−22	0.015	13
Temporal pole		5.12	−40	14	−24	0.018	21
Caudate	L	5.10	−14	10	10	0.020	26

* Family wise error-corrected (voxel-level), voxel-by-voxel analysis at *p* < 0.05. Hemispheres are indicated as L = left and R = right. MNI, Montreal Neurological Institute.

**Table 3:** Brain areas with significantly greater activation in the choice than control condition during the ER or staying part.

Area	Hemisphere	*t* Value	MNI Peak Coordinates			*p* Value	*k*
			x	y	z		
Supplementary motor area *	L	7.52	−6	10	64	<0.001	674
Precentral gyrus	L	6.34	−46	2	50	<0.001	193
Frontal operculum	L	5.36	−48	14	−6	0.001	321
Opercular part of the inferior frontal gyrus		5.21	−50	20	10	0.002	
Middle temporal gyrus	L	5.03	−54	−26	−8	0.005	83
Caudate	L	4.92	−16	10	12	0.008	23

* Family-wise error-corrected (voxel-level), voxel-by-voxel analysis at *p* < 0.05. Hemispheres are indicated as L = left and R = right. MNI, Montreal Neurological Institute.

**Table 4:** Brain areas with greater activation in the choice than forced condition during the ER or staying part.

Area	Hemisphere	*t* Value	MNI Peak Coordinates			Uncorrected *p* Value	*k*
			x	y	z		
Supramarginal gyrus *	R	3.87	54	−40	50	<0.001	117
Superior parietal lobule	R	3.39	42	−46	56	0.001	
Postcentral gyrus	L	3.24	−64	−16	24	0.001	2

* Voxel-by-voxel analysis without correction for multiple comparisons. Hemispheres are indicated as L = left and R = right. MNI, Montreal Neurological Institute.

**Table 5:** Brain areas with significantly greater activation in the forced than choice condition during the ER or staying part.

Area	Hemisphere	*t* Value	MNI Peak Coordinates			*p* Value	*k*
			x	y	z		
Calcarine cortex *	R	9.79	12	−78	0	<0.001	837
	L	6.95	−12	−80	0	<0.001	
Fusiform gyrus	L	5.77	−30	−78	−16	0.002	55
Fusiform gyrus	R	4.98	32	−74	−10	0.029	4

* Family-wise error-corrected (voxel-level, voxel-by-voxel analysis at *p* < 0.05. Hemispheres are indicated as L = left and R = right. MNI, Montreal Neurological Institute.

**Table 6:** Common brain areas with significant activation in both the choice and forced conditions during the ER or staying part.

Area	Hemisphere	*t* Value	MNI Peak Coordinates			*p* Value	*k*
			x	y	z		
Supplementary motor area *	L	7.97	−6	12	62	<0.001	469
Middle frontal gyrus	L	7.22	−44	4	52	<0.001	182
Opercular part of the inferior frontal gyrus	L	5.69	−50	20	12	0.002	188
Frontal operculum	L	5.60	−50	18	−6	0.003	
Opercular part of the inferior frontal gyrus	L	5.41	−52	20	4	0.007	
Middle temporal gyrus	L	5.17	−52	−32	−6	0.016	45
	L	5.02	−54	−24	−8	0.025	
Caudate	L	5.08	−14	10	10	0.021	11

* Family-wise error-corrected (voxel-level), voxel-by-voxel analysis at *p* < 0.05. Hemispheres are indicated as L = left and R = right. MNI, Montreal Neurological Institute.

## Data Availability

The raw data supporting the conclusions of this article will be made available by the authors without undue reservation.

## Supplementary Materials

The following supporting information can be downloaded at: https://www.mdpi.com/article/10.3390/brainsci14111077/s1, Table S1: Contrast of time 2 and time 1 in each condition, Table S2: Brain areas with significantly greater activation in the forced than control condition during the ER or staying part, considering individual difference in the tendency to choose refocus on planning; Table S3: Brain areas with significantly greater activation in the choice than control condition during the ER or staying part, considering individual difference in the tendency to choose refocus on planning; Table S4: Common brain areas with significant activation in both the choice and forced conditions during the ER or staying part, considering individual difference in the tendency to choose refocus on planning; Table S5: Brain areas with significantly greater activation in the forced than choice condition during the ER or staying part, considering individual difference in the tendency to choose refocus on planning.
