# Supplementary material for: Self-Choice Emotion Regulation Enhances Stress Reduction: Neural Basis of Self-Choice Emotion Regulation
Source: Brain Sci. 2024 Oct 28;14(11):1077. doi: 10.3390/brainsci14111077 (PMC11591930; doi:10.3390/brainsci14111077)
Supplement: Supplementary file 1 [file brainsci-14-01077-s001.zip › brainsci-3254364-supplementary.pdf]

**Supplementary Materials:**

**Table S1.** Contrast of time 2 and time 1 in each condition.

| Contrast | $\beta$ Estimate | Std. Error | 95% CI lower | 95% CI upper | $t$ value | df |
|---|---|---|---|---|---|---|
| choice time2 - choice time1 | −0.766 | 0.057 | −0.878 | −0.654 | −13.415 | 2651.078 |
| forced time2 - forced time1 | −0.698 | 0.057 | −0.81 | −0.586 | −12.223 | 2651.078 |
| control time2 - control time1 | −0.294 | 0.057 | −0.406 | −0.182 | −5.159 | 2651.078 |

**Table S2.** Brain areas with significantly greater activation in the forced than control condition during the ER or staying part, considering individual difference in the tendency to choose refocus on planning.

| Area | Hemisphere | $t$-value | MNI peak coordinates | | | $p$-value | $k$ |
|---|---|---|---|---|---|---|---|
| | | | x | y | z | | |
| Supplementary motor area * | L | 6.82 | −6 | 12 | 62 | <0.001 | 784 |
| | | 5.79 | −6 | 20 | 50 | <0.001 | |
| | | 5.23 | −4 | 24 | 40 | 0.002 | |
| Middle frontal gyrus | L | 6.37 | −46 | 4 | 52 | <0.001 | 318 |
| Opercular part of inferior frontal gyrus | L | 5.84 | −50 | 20 | 18 | <0.001 | 596 |
| Frontal operculum | L | 5.15 | −50 | 18 | −6 | 0.003 | |
| Opercular part of inferior frontal gyrus | L | 5.48 | −50 | 20 | 4 | 0.005 | |
| Middle temporal gyrus | L | 5.38 | −52 | −38 | −4 | 0.001 | 224 |
| | | 4.69 | −54 | −24 | −8 | 0.023 | |
| Fusiform gyrus | L | 5.25 | −30 | −44 | −14 | 0.002 | 23 |
| Temporal pole | L | 5.20 | −50 | 8 | -22 | 0.014 | 13 |
| Temporal pole | L | 5.12 | −40 | 14 | −24 | 0.016 | 21 |
| Caudate | L | 5.10 | −14 | 10 | 10 | 0.017 | 28 |

* Voxel by voxel analysis at $p < 0.05$ Family wise error-corrected (voxel-level). Hemispheres are indicated as L = left, R = right. MNI, Montreal Neurological Institute.

**Table S3.** Brain areas with significantly greater activation in the choice than control condition during the ER or staying part, considering individual difference in the tendency to choose refocus on planning.

| Area | Hemisphere | $t$-value | MNI peak coordinates | | | $p$-value | $k$ |
|---|---|---|---|---|---|---|---|
| | | | x | y | z | | |
| Supplementary motor area * | L | 7.58 | −6 | 10 | 64 | <0.001 | 689 |
| Precentral gyrus | L | 6.39 | −46 | 2 | 50 | <0.001 | 202 |
| Frontal operculum | L | 5.42 | −48 | 14 | −6 | 0.001 | 338 |
| Opercular part of inferior frontal gyrus | | 5.27 | −50 | 20 | 10 | 0.002 | |
| Middle temporal gyrus | L | 5.10 | −54 | −26 | −8 | 0.004 | 96 |
| Caudate | L | 4.99 | −16 | 10 | 12 | 0.006 | 29 |

* Voxel by voxel analysis at $p < 0.05$ Family wise error-corrected (voxel-level). Hemispheres are indicated as L = left, R = right. MNI, Montreal Neurological Institute.

**Table S4.** Common brain areas with significant activation in both the choice and forced conditions during the ER or staying part, considering individual difference in the tendency to choose refocus on planning

| Area | Hemisphere | *t*-value | MNI peak coordinates | | | *p*-value | *k* |
|---|---|---|---|---|---|---|---|
| | | | x | y | z | | |
| Supplementary motor area * | L | 6.82 | −6 | 12 | 62 | <0.001 | 477 |
| Middle frontal gyrus | L | 6.34 | −44 | 4 | 52 | <0.001 | 190 |
| Opercular part of inferior frontal gyrus | L | 5.25 | −50 | 20 | 12 | 0.002 | 206 |
| Frontal operculum | L | 5.15 | -50 | 18 | −6 | 0.003 | |
| Opercular part of inferior frontal gyrus | L | 5.00 | −52 | 20 | 4 | 0.006 | |
| Middle temporal gyrus | L | 4.83 | −52 | −32 | −6 | 0.012 | 54 |
| | L | 4.69 | −54 | −24 | −8 | 0.023 | |
| Caudate | L | 4.76 | −14 | 10 | 10 | 0.017 | 12 |

* Voxel by voxel analysis at $p < 0.05$ Family wise error-corrected (voxel-level). Hemispheres are indicated as L = left, R = right. MNI, Montreal Neurological Institute.

**Table S5.** Brain areas with significantly greater activation in the forced than choice condition during the ER or staying part, considering individual difference in the tendency to choose refocus on planning.

| Area | Hemisphere | *t*-value | MNI peak coordinates | | | *p*-value | *k* |
|---|---|---|---|---|---|---|---|
| | | | x | y | z | | |
| Calcarine cortex * | R | 9.79 | 12 | −78 | 0 | <0.001 | 799 |
| | L | 6.95 | −12 | −80 | 0 | <0.001 | |
| Fusiform gyrus | L | 5.77 | −30 | −78 | −16 | 0.003 | 47 |
| Fusiform gyrus | R | 4.98 | 32 | −74 | −10 | 0.042 | 2 |

* Voxel by voxel analysis at $p < 0.05$ Family wise error-corrected (voxel-level). Hemispheres are indicated as L = left, R = right. MNI, Montreal Neurological Institute.
